# Design of halochromic cellulosic materials and smart textiles for continuous wearable optical monitoring of epidermal pH

**DOI:** 10.1007/s00604-025-07259-x

**Published:** 2025-06-04

**Authors:** Gerhard J. Mohr, Petar Kassal, Iva Žuvić, Krzysztof K. Krawczyk, Matthew D. Steinberg, Ivana Murković Steinberg

**Affiliations:** 1 Joanneum Research Forschungsgesellschaft mbH – Materials, Franz-Pichler-Straße 30, Weiz, A-8160 Austria; 2 Faculty of Chemical Engineering and Technology, University of Zagreb, Marulićev Trg 19, Zagreb, 10000 Croatia; 3 GoSense Wireless Ltd, Cambridge, CB23 6 FN UK

**Keywords:** Wearable pH sensors, Colorimetric sensors, Functional cellulose materials, Vinylsulfonyl chemistry, Wearable optoelectronics, Epidermal pH

## Abstract

The direct *on-body* monitoring of sweat pH and skin pH can provide vanguard information about a person’s health status and overall fitness or wellbeing. Sweat pH in particular is a readily accessible biomarker that may be determined with low-cost, non-invasive epidermal patches. With this in mind, we have created a diverse set of wearable halochromic materials, suitable for real-time and reversible monitoring of epidermal pH. A range of cellulosic materials in the form of nanocrystals, microcellulose particles, transparent cellulose films and paper were covalently functionalised with a 2-hydroxyethylsulfonyl azo pH indicator dye by vinylsulfonyl conjugation. Comparative analysis of the photo-physical and analytical properties was performed, and the apparent acid–base equilibrium constants — p*K*_*app*_ values of the immobilised indicator — ranging from 5.6 for cellulose paper to 6.9 for nanocellulose particles, cover the physiological range of epidermal sweat and skin pH. A polyester textile — typical of modern sports apparel — was subsequently functionalised with the biocompatible hydrogel containing the halochromic cellulose microparticles. The colour change of the smart textile was found to be fully reversible (*RSD* = 0.14%) and its pH response highly stable for over 280 min upon continuous exposure to pH buffers (signal change of 0.16%). The halochromic textile was characterised with custom-made low-power wearable optoelectronics combining a reflectometer and real-time data logger, and the system bench-marked against a calibrated laboratory glass pH electrode and meter. The wearable optoelectronic and halochromic textile system was able to continuously monitor pH in buffer solutions over the physiologically relevant range with an accuracy of 0.18 pH and precision of 0.05 pH units.

**Graphical abstract:**

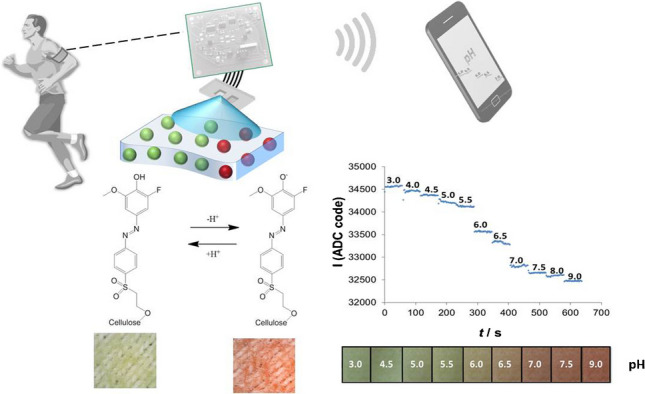

**Supplementary Information:**

The online version contains supplementary material available at 10.1007/s00604-025-07259-x.

## Introduction

Interest in wearable (bio)chemical sensors is growing rapidly as these devices can provide insight into a person’s health status through non-invasive monitoring of biochemical markers in fluids such as sweat, tears, saliva and interstitial fluid (ISF). Key biomarkers can reveal different aspects of a person’s physiology: from hydration and physical fatigue, to mental stress and disease [1, 2]. Sweat is the most explored of these biofluids, with major research effort currently directed toward development of epidermal chemical sensors, in particular electrochemical and optical sensors for the determination of sweat electrolytes and pH [3–6]. Electrochemical sweat sensors were amongst the earliest wearable devices to appear in the literature [7], whereas wearable photonic (optical) sensors have more recently exploded onto the scene [8–10]. The interest in optical solutions is driven by advances in biocompatible photonic (nano)materials and wearable optoelectronics, combined with the intrinsic non-invasive and contactless nature of light interactions. 

Non-invasive epidermal pH sensors are usually designed to monitor the pH of sweat. Sweat pH lies between 5 and 7 and is considered an index of hydration state, which is useful for both medicine and fitness/wellness [11]. Furthermore, sweat pH can be correlated to sweat electrolyte levels [12, 13], as well as to the onset of metabolic alkalosis, and can thus be used as a proxy for monitoring muscle fatigue during exercise [14, 15]. In addition to sweat pH, there are two other skin pH-related biomarkers, mainly used in dermatology and skin physiology to assess healthy and diseased skin — *skin surface pH* and *skin buffer capacity* [16]. Normally, human skin surface pH is acidic and ranges between 4.2 and 6.1. The *apparent pH of skin* is most commonly measured with a planar glass electrode (or less often, optically) according to a defined protocol. Measurements are made for routine skin health diagnostics [17] or for studying the therapeutic effect of treatment for various skin conditions, such as atopic dermatitis [18]. Finnegan et al. introduced a novel wearable method for the indirect determination of *skin surface pH* via volatile ammonia emission using colourimetric sensors that non-invasively monitor skin physiology, without the need for microneedles to access ISF or sweat harvesting from the skin surface [19].

Wolfbeis and co-workers extensively reviewed the field of optical pH sensing materials, thereby providing both a valuable reference source and inspiration for the development of new photonic materials and the design of sensing schemes for integration with wearable devices [20].

Wearable pH sensitive materials should be biocompatible, flexible and have a reversible response for long-term monitoring [21].

The potential of integrating sensors with fabrics to form optical pH-responsive (halochromic) textiles has long been recognised [22]. De Clerck’s group used different pH indicator dyes with conventional dyeing and electrospinning techniques to form halochromic textiles based on cotton [23] and polycaprolactone/chitosan [24] fabrics, respectively. Other fabric-based examples include surface modification of cotton fibres with chitosan to immobilise Bromocresol green [25], cotton modified by 3-glycidoxypropyltrimethoxysilane with a sol–gel dye immobilisation process [11], and polyester fabrics modified with ionophore-based reversible optodes [26]. Colourimetric materials intended for integration with pH-sensitive clothing have been designed including curcumin and thermoplastic-polyurethane (C-TPU) electrospun-fibres deposited on various fabric substrates [27], heterocyclic azo dyes with single and two D–**π**–A systems [28], and a naphthalimide-rhodamine probe, both covalently immobilised on cotton [29].

Popular, microfluidic sweat-wicking wearable patches [30] and devices using chrono-sampling methods for the sequential paper-based colourimentrc pH measurement overcome problem of irreversible chemistry [31], whilst screen printing of wax inks on cotton/elastane fabrics can deliver low-cost and biocompatible microfluidic systems for colourimetric analysis of pH and urea in sweat [32].

Some of the first integrated solutions — combining materials and instrumentation — for wearable optical pH and ions monitoring using halochromic textiles were developed by Diamond’s group [33]. Caldara et al. developed a wearable pH sensor based on a cotton fabric treated with an organically modified silicate with covalently immobilised litmus [34], and we reported a mobile IoT analytical fluorimetry platform demonstrating chloride determination via a paper-immobilised quinine sulphate [35]. More recent examples include a wearable fluorimetric platform in the format of sweat sensor sticker with integrated readout module using transparent chitin nanopaper [36], and a wireless wristband for the continuous colourimetric monitoring of sweat pH based on a functionalised microfluidic cloth analytical device (μCAD) [37]. Rogers and co-workers over many years have developed a range of on-skin, epifluidic platforms with integrated wireless electronics and colourimetric chemical reagents for capture, storage, and chemical analysis of sweat. In their recent work, they present a wearable microfluidic band to measure sweat biochemistry of muscles which monitors both pH and lactate concentration in sweat, as indicators of muscle fatigue [38].

Cellulose as a natural biopolymer is ideally suited for use as a substrate material in wearable skin-sensing applications. Many pH responsive optical materials based on cellulose fibres and nanocrystals of different formats such as hydrogels, aerogels, films, papers, and fabrics have therefore been devised [39]. The specific immobilisation method chosen to incorporate optically responsive molecules into/onto substrate materials however is key to ensuring a final hybrid material that retains adequate sensing properties (range, sensitivity, reversibility, response time and stability/longevity) [40]. Moreover, substrate materials for wearable applications have to be biocompatible, flexible, comfortable, and often may need to be compatible with other sports apparel and possibly even with ubiquitous consumer products such as fitness trackers and smart watches. Covalently immobilised indicators on natural biopolymers — such as cellulose — offer the right combination of functional properties. Covalent immobilisation however often involves tedious synthetic procedures and/or use of environmentally unfriendly processes and toxic chemicals. To address the need for simpler and greener fabrication chemistry, Mohr and Wolfbeis [41] introduced a one-pot method for the covalent immobilisation of azo pH indicators — known for their photochemical stability and brightness — onto cellulose substrates using vinylsulfonyl conjugation. This method has since been exploited in a variety of cellulose-based sensors and textiles [37, 42–45].

**Fig. 1:**
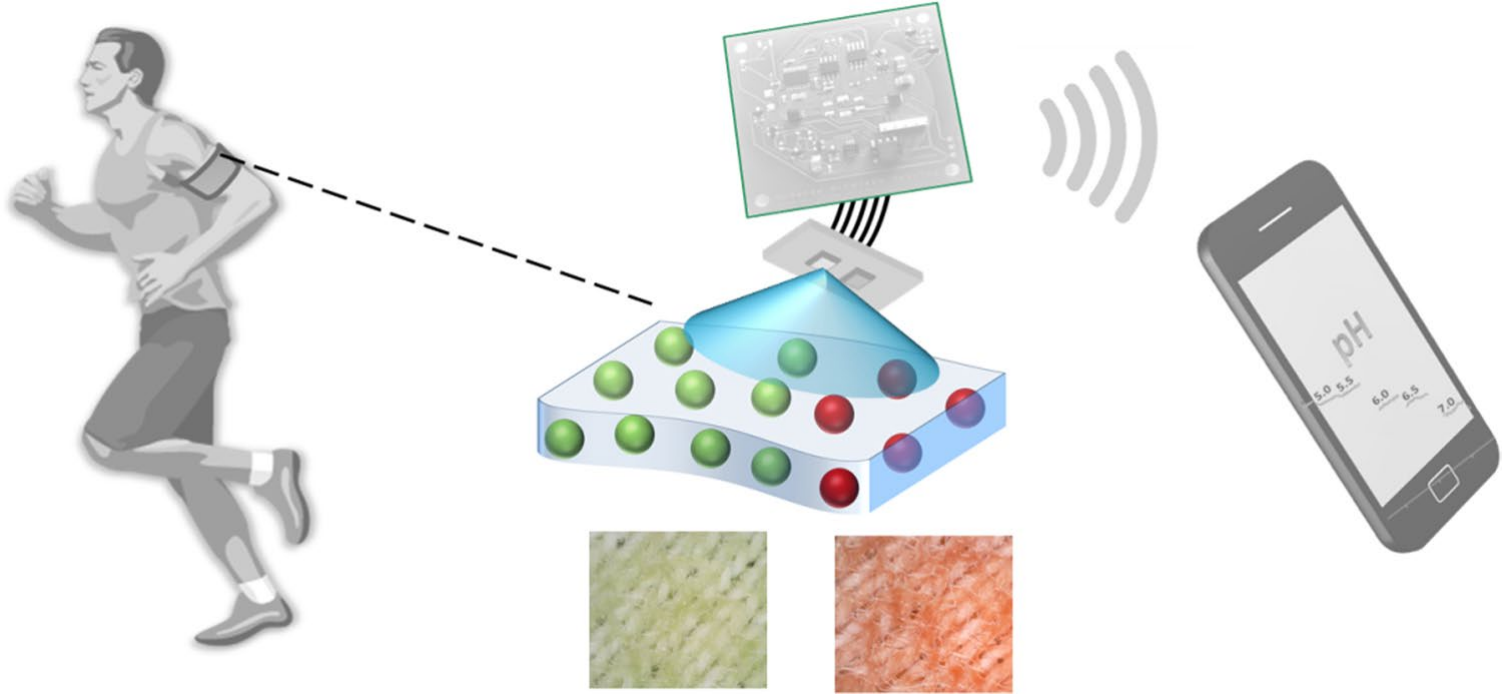
Wearable use-case scenario based on halochromic smart textile for monitoring sweat pH

In this present work, we fabricated a range of cellulose-based materials — cellulose nanocrystals, microcellulose particles, transparent cellulose films and paper — covalently functionalised with a 2-hydroxyethylsulfonyl azo pH indicator dye to create a diverse set of new halochromic materials. The applicability of these new materials to pH monitoring is evaluated and presented, and a typical wearable use-case scenario is proposed. Furthermore, a polyester textile shirt, typical of modern sports apparel, was successfully functionalised with hydrogel containing halochromic cellulose microparticles. The pH sensing performance of the smart polyester textile was characterised with a low-power wearable optoelectronic detector, bench-marked against a laboratory pH meter (Fig. 1).

The development of our wearable sensing materials has been guided by the four key objectives recently posited by Gao and co-workers [8]:

(1) optimise the photonic analytical method, (2) engineer materials that support photonic and wearable functionality, (3) implement scalable fabrication processes for (future) miniaturisation, mass production, and versatile applications, and (4) integrate advanced power management strategies for effective real-world performance.

## Materials and methods

### Reagents and materials

The pH indicator dye 2-fluoro-4-[4-(2-hydroxyethanesulfonyl)-phenylazo]−6-methoxyphenol, **1** [44, 46] was from Joanneum Research Forschungsgesellschaft mbH (Austria). The inert dye disodium 1-amino-9,10-dioxo-4-[3-(2-sulfonatooxyethylsulfonyl)-anilino]-anthracene-2-sulfonate (Remazol Brilliant Blue R, RBBR), Sigmacell Cellulose Type 20 particles and Whatman cellulose paper sheets Grade 1 Chr were from Aldrich (Austria). Wide pH ranging buffer solutions were prepared from citric acid, boric acid, phosphoric acid, sodium hydroxide, and hydrochloric acid, all obtained from Kemika (Croatia). Artificial sweat was prepared according to the recipe previously described [4] and contained 50 mM NaCl, 6 mM KCl, 5 mM NH_4_Cl, 0.08 mM MgCl_2_, 2.6 mM NaHCO_3_ and 0.04 mM Na_2_HPO_4_. Regenerated cellulose layers Innovia 25 NP with a nominal thickness of 25 μm were from Futamura (Japan). Dry cellulose nanocrystals (CN), produced by sulfuric acid hydrolysis of wood pulp, were kindly provided by CelluForce (Montreal, Canada) with nanocrystal average length and diameter of 100 and 5 nm, respectively. HydroMed™ D4 polyurethane-based hydrogel was from Cardiotech (USA). Printperfekt 226 EC and Printperfekt Base FF were kindly provided by CHT Austria R. Beitlich GmbH. The polyester shirt was from Xpres (UK).

### Preparation of coloured cellulose particles and sensor layers

#### Cellulose materials CF − 1* and CP − 1*

In a typical immobilisation procedure [44], each 100 mg of **1** was treated with 1.0 g of concentrated sulfuric acid for 30 min at room temperature. This converted the 2-hydroxyethylsulfonyl group of the indicator dye into the sulfonate.

Then, the mixture was poured into 900 mL of distilled water and neutralised with 1.6 mL of a 30% sodium hydroxide solution. At this stage, 80 mg of the inert dye RBBR (already a sulfonate) was added to the solution of 1 to provide the green–red colour change. Then, 25.0 g of sodium carbonate in 100 mL of water and 5.2 mL of a 30% sodium hydroxide solution were added. The cellulose substrate (either the Innovia cellulose film or the Whatman cellulose paper) were placed into this solution. Upon addition of sodium hydroxide and sodium carbonate, the dye sulfonates were converted into vinylsulfonyl derivatives and coupled via Michael addition to the hydroxyl groups of cellulose. After 60 min, the coloured materials were removed from the dyeing bath and washed with copious amounts of distilled water.

#### Cellulose nanoparticles NC − 1

Firstly, 5 g of dry cellulose nanoparticles (NC) was dispersed into 70 mL of distilled water and stirred for one hour. Then, 20 mg of **1** was treated with 0.25 g of concentrated sulfuric acid for 30 min at room temperature, subsequently mixed with 40 mL of water, and made alkaline by addition of 1.75 mL of 30% sodium hydroxide solution. Then, this solution was poured into the dispersion of cellulose nanoparticles and stirred rigorously for one hour. Afterwards, the coloured cellulose nanoparticles NC-**1** were treated with 2 mL of 6 N HCl to terminate the immobilisation reaction, followed by addition of ethanol to induce precipitation of NC-**1**. The nanoparticles were centrifuged using an EBA 21 (Hettich, Germany) at 5000 rpm for 10 min, the supernatant solution discarded and the preprecipitate was re-dispersed in ethanol to remove any unbound dye from the nanoparticles. This purification procedure was repeated six times. Afterwards, NC-**1** nanoparticles were stored in the presence of ethanol.

#### MC − 1*

Firstly, 100 mg of **1** was treated with 1.0 g of concentrated sulfuric acid for 30 min at room temperature. Then, the solution was poured into 200 mL of distilled water containing 50.0 g of Sigmacell cellulose microparticles and neutralised with 1.6 mL of a 30% sodium hydroxide solution. At this stage, 80 mg of the inert dye RBBR (already a sulfonate) was added to the solution. Then, 25.0 g of sodium carbonate in 100 mL of water and 5.2 mL of a 30% sodium hydroxide solution were added as well. After 60 min, the particles were filtrated using a Buchner funnel and were washed repeatedly with distilled water and acetone to remove salts and unbound dyes.

#### MC − 1* in hydrogel@polyester (smart textile)

The dry MC-1* were then dispersed into the 5% polymer solution of HydroMed™ D4 in aqueous ethanol (ethanol/water = 9:1) and stirred overnight. The well stirred dispersion was pipetted onto a commercial polyester shirt and spread using a knife-coating blade to form a thin pH sensitive layer with dimensions 15 mm × 15 mm.

### Characterisation experiments

#### Spectral characterisation

A Shimadzu UV-1280 UV–Vis spectrophotometer (Shimadzu Scientific Instruments, Kyoto, Japan) was used for recording absorbance spectra. Thin cellulose films were cut to fit standard cuvette, placed against the cuvette wall and exposed to buffer solutions of different pH.

A modular fibre optic reflectance spectrometer (FLMS03018, Ocean Optics, USA) with a balanced deuterium hydrogen light source (DH2000 BAL, Ocean Optics, USA) was used to measure reflectance spectra of non-transparent cellulose materials.

#### Dynamic response

The sensor textile layers were evaluated in a custom-made flow cell to determine the dynamic pH response. A modular fibre optic reflectance spectrometer was used to measure the reflectance at 525 nm. The buffer solutions were continuously pumped into the flow cell using a peristaltic pump (Masterflex C/L, model 77120–62, Cole-Parmer, USA) at a flow rate of 0.12 mL/min. The reflectometric probe was placed under the flow-through cell facing the textile, measuring the sensor response to different pH values.

#### Wireless experiments

The polyester shirt sensor was read with an ultra-low power data logger designed for optical sensing (OptiTag, GoSense Wireless Ltd, UK) that we have reported elsewhere [43]. Briefly, the battery powered data logger is built around a low-energy integrated circuit (MLX90129, Melexis BV, Belgium) which supports data communication with mobile devices via NFC. The data logger interrogates the halochromic sensor with a miniature optoelectronic reflectance probe. The probe — matched to the spectroscopic characteristics of **1** — consists of a cyan surface mount LED (HSMK-A100-S00 J1, Avago/Broadcom, USA) and a photodiode (BPW34S, Osram Opto Semiconductors, Germany). The LED and PD are controlled by the data logger during sample acquisition at an adjustable sample rate.

In a typical experiment, 1 mL of buffer solution was pipetted onto the textile. After 2 min of equilibration, measurements with the data logger were initialised using a USB-powered desktop RFID reader (Proxima RF Inc, USA) or by mobile phone running a custom-designed Android application (eTag Reader, GoSense Wireless Ltd, UK). The optical probe was placed above the pH sensitive film and 30 reflectance data points were recorded over a period of 1 min. After each 1-min measurement epoch, the textile sensor layers were thoroughly rinsed with deionised water to remove any remaining buffer solution, after which the procedure was repeated with new buffers. The recorded data was transferred by NFC from the data logger to the smartphone and analysed off-line with Microsoft Excel. Results were correlated against pH values obtained with a calibrated laboratory pH meter (Iskra MA5740, Metrel d.d., Slovenia) and combination pH electrode (BlueLine 17 pH, Schott AG, Germany).

## Results and discussion

### Choice of cellulose materials and immobilisation method

**Fig. 2:**
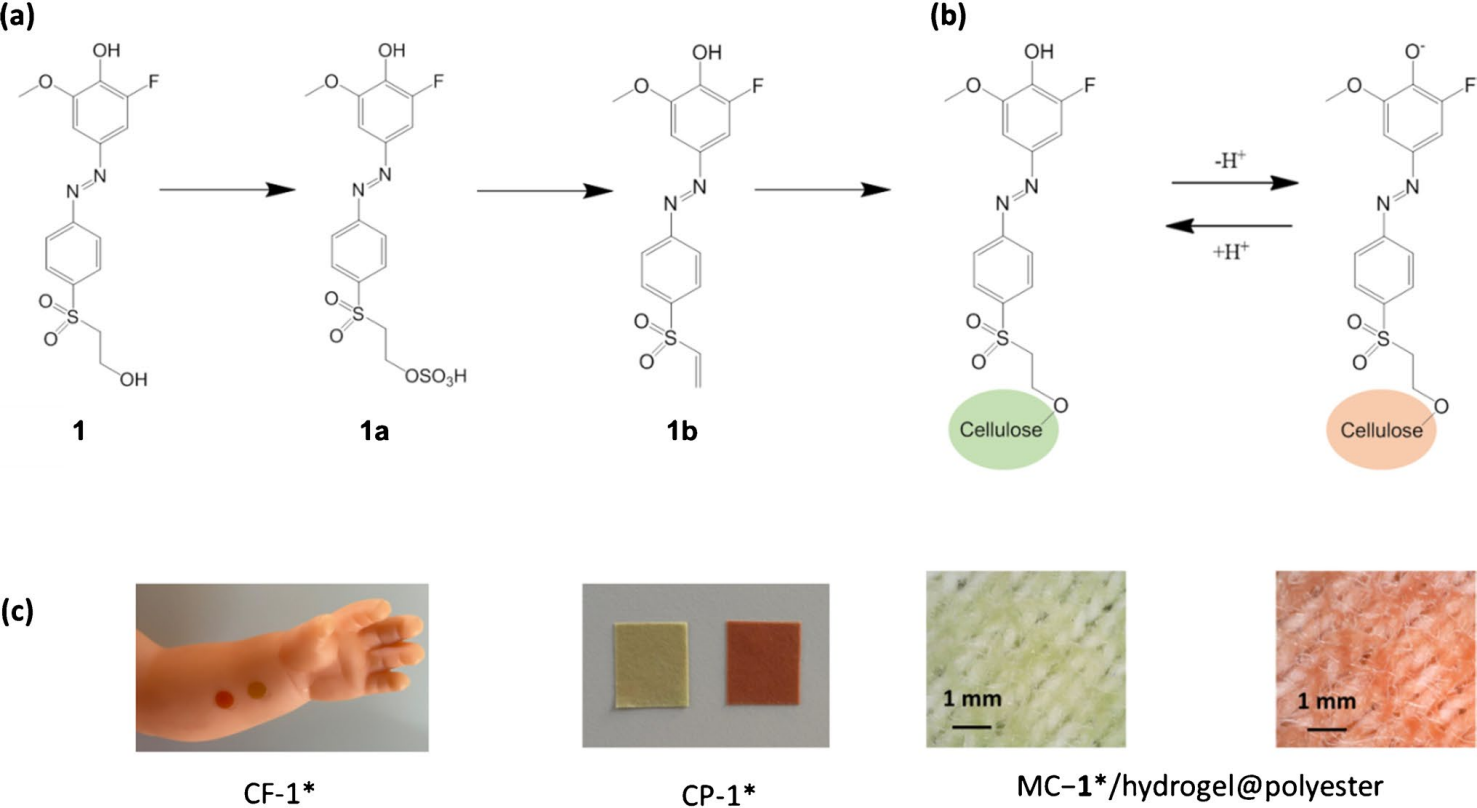
**a** Molecular structure of acidochromic dye **1** and its conversion to **1a** and **1b** during immobilisation reaction based on vinylsulfoyl chemistry. **b** Acid–base equilibrium of the immobilised dye. **c** Photographs of different formats of tested cellulosic materials: cellulose foils (CF − 1*) shown in a potential use-case scenario, paper CP − 1* and the textile MC − 1*/hydrogel@polyester. Materials are exposed to pH 5 (green) and pH 7 (red), (MC − 1*/hydrogel@polyester shown as microscopy images revealing the woven structure of polyester yarns)

A pH sensitive reactive azo dye, 2-fluoro-4-[4-(2-hydroxyethanesulfonyl)-phenylazo]−6-methoxyphenol, **1** (Fig. 2a) was selected as the pH indicator candidate for covalent immobilisation on a range of cellulose materials for continuous epidermal pH sensing [46]. Its p*K*_a_ value of around 6 is well suited to cover the dynamic range for skin surface and sweat sensing applications, typically between pH 5 and 7. Also, cytotoxic studies of cellulose particles and films functionalised with dye **1** were previously performed according to ISO 10993, where it was found that neither the undiluted eluate nor direct contact with the MRC-5 cells caused any decrease in cell viability [47], which makes them promising candidates for direct epidermal applications.

Covalent immobilisation of **1** is based on a simple one-pot vinylsulfonyl chemistry, schematically shown in Fig. 2a [46]. Typically, cellulosic material is immersed into the dyeing bath in which dye **1** has been firstly converted into sulfonate **1a** (in acidic conditions), followed by conversion into a chemically reactive vinylsulfonyl derivative **1b,** under basic conditions. The vinylsulfonyl groups of **1b** react with hydroxyl groups of the cellulose materials via Michael addition providing a covalent attachment of the pH sensitive dye molecule onto the cellulosic material Fig. 2b.

The pH indicator **1** shows a visible colour change from yellow to red in going from acidic to basic form. In order to provide a visually stronger colour change on cellulose substrates, a pH insensitive vinylsulfonyl blue dye (RBBR) was co-immobilised with **1** which resulted in an acid–base colour change from green to red (Fig. 2b). We have demonstrated this approach previously in fabrication of pH sensitive cellulose films and cotton-based textiles [44]. The pH insensitive absorption band of the material in the red spectral region (around 650 nm) also provides a reference point for instrumental optical pH determination.

### Functionalised cellulose materials

In this work, the following cellulosic materials were fabricated and evaluated for potential use in epidermal pH monitoring: cellulose nanocrystals, microcellulose fibres, transparent cellulose films and cellulose chromatography paper. All cellulosic materials are commercially available and were functionalised with **1** using vinylsulfonyl chemistry, as described.

The pristine cellulose materials were obtained via different dissolution and regeneration routes and from different plant sources, which generally may affect their chemical and morphological properties including efficiency for covalent grafting of functional molecules on them, wicking capacity, transparency, etc. [39]. A summary of the materials, their general properties and functionalisation details are shown in Table 1.

**Table 1:** Functionalised cellulose materials and their characteristics

Material	Properties
NC − **1**	Cellulose nanocrystals (NC) produced by sulfuric acid hydrolysis of wood pulp, with average length and diameter of 100 and 5 nm, respectively; functionalised with **1**, characterised in a form of colloidal aqueous dispersions
CF − 1*	Transparent cellulose films (CF) co-functionalised with **1** and RBBR 25 μm thick, prepared from regenerated cellulose; potential for direct determination of pH on skin surface
CP − 1*	Cellulose paper (CP) co-functionalised with **1** and RBBR, produced from the highest quality cotton linters with no additives; passive microfluidic capability—linear flow rate 130 mm/30 min, not transparent, thickness 0.18 mm
MC − 1*	Microcellulose particles (MC), produced from cotton linter, average fibre size 20 μm, co-functionalised with **1** and RBBR; embedded in hydrogel for functionalisation of polyester textile

Images of the functionalised cellulosic materials in their respective acidic and basic forms at pH 5 and pH 7, are shown in Fig. 2c (CF − **1**, CP − 1* and MC − 1*), and in Fig. S1 (NP-**1**).

### Photophysical characterisation and pH response of materials

**Fig. 3:**
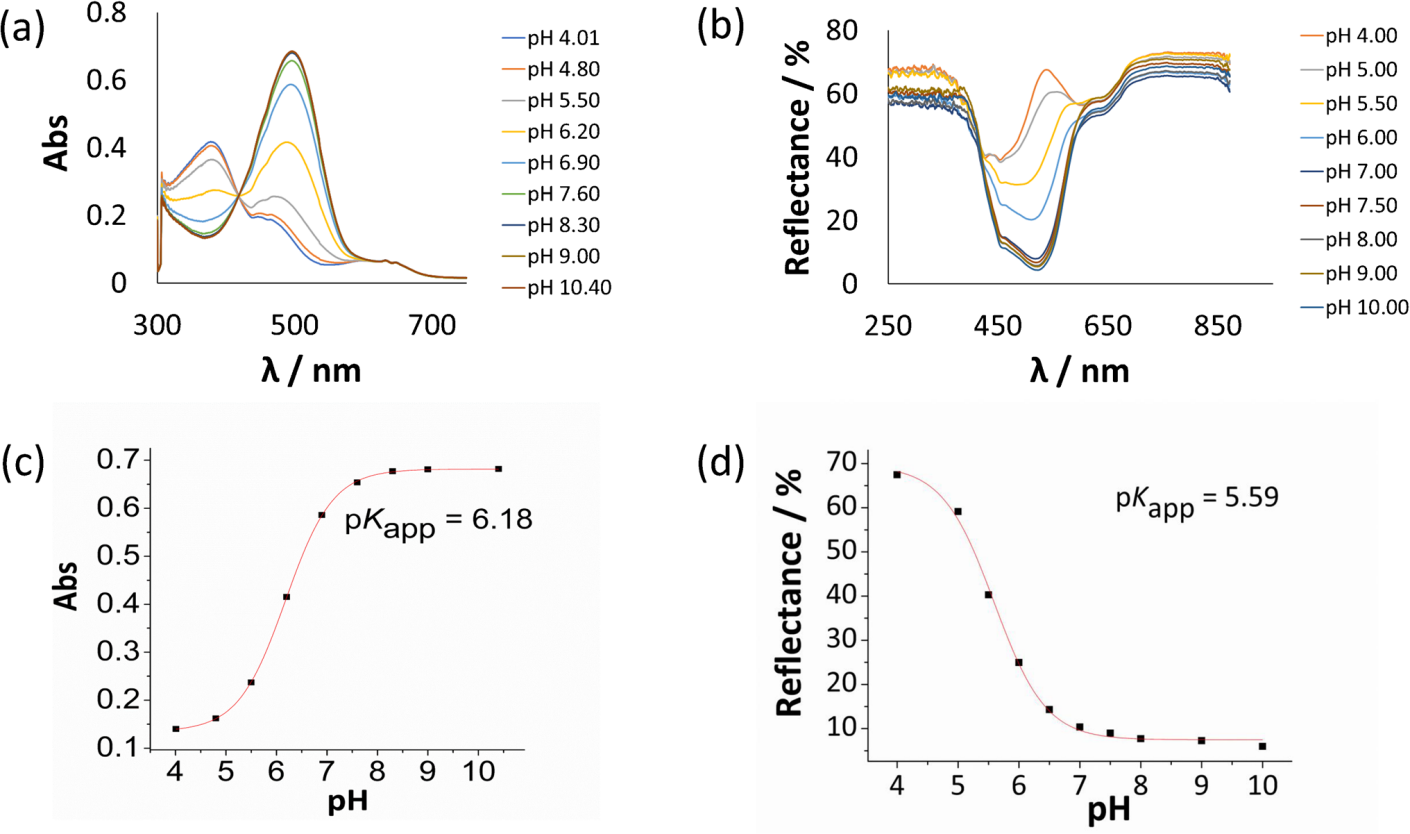
**a** Spectroscopic pH titration—absorption spectra of transparent cellulose film CF − 1* and **b** reflectance spectra of functionalised cellulose paper CP − 1*, **c** and **d** their respective pH titration plots and calculated p*K*_*app*_ values

All modified cellulosic materials were firstly characterised spectroscopically on exposure to buffer solutions in the pH range 4 to 10. The UV–Vis absorption spectroscopy was used for transparent films (CF-1*), and for nanocellulose dispersions (NC-**1**), as shown in Fig. 3a and Fig. S1, respectively. UV–vis reflectance spectra of the non-transparent material, cellulose paper, are shown in Fig. 3b.

The respective pH response curves were obtained by plotting the absorbance or reflectance intensity of the maximum/minimum of deprotonated form of the dye (around 500 nm) against the pH value. The experimental data were fitted to sigmoid Boltzmann curve and the respective, apparent acid–base equilibrium constants p*K*_*app*_ of each material were determined, as shown in Fig. 3c and 3d for CF-1* and NC-**1**, respectively.

A comparison of the photophysical and pH sensing properties (p*K*_*app*_ values) of dye **1** dissolved in solution and immobilised on cellulosic materials is presented in Table 2.

**Table 2:** Summary and comparison of photophysical and pH sensing properties of functionalised cellulosic materials based on dye **1** (*measured in buffer–methanol, volume ratio *Φ* = 1:1 [46])

Sample	*λ*_max_ acid/*λ*_max_ base	p*K*_app_
**1** in solution*	Absorbance, 372 nm/487 nm	6.8
NC − **1**	Absorbance 378 nm/500 nm	6.9
CF − 1*	Absorbance 378 nm/492 nm	6.2
CP − 1*	Reflectance, 535 nm (basic form)	5.6

Spectral characterisation revealed small changes in wavelengths of absorption maxima of immobilised **1**, slightly more pronounced in base (anionic) form. This is not unexpected given molecular/microstructural surface differences of cellulosic materials. However, for sensing applications those differences are not relevant. The coloured crystalline nanocellulose particles are not fully transparent in aqueous dispersion, and the spectra show an increasing absorbance caused by Mie scattering in going from 800 to 300 nm The spectral changes upon exposure to buffers of different pH are superimposed on the absorbance caused by light scattering and can clearly be distinguished visually and spectroscopically (Fig. S1).

The p*K*_*app*_ values of the materials change in the range from 5.6 for the cellulose paper, to 6.9 for nanocellulose particles.

It has been shown for similar types of dye, also neutral in acidic form like **1**, that p*K*_*app*_ values measured in methanol–water mixtures are approximately one unit higher than those measured in aqueous buffers [46]. We can conclude that deprotonation of **1** grafted on cellulose materials appears at lower pH values (making it stronger acid) in comparison with in methanol–water mixtures (p*K*_app_ = 6.8). The exception is nanocellulose where the shift of p*K*_*app*_ value to 6.9 can be attributed to sulphate groups on the nanocellulose surface which resulted from the production process which uses concentrated sulphuric acid for hydrolysis of wood pulp. The sulphate groups restrict the deprotonation of the dye and shift the p*K*_*app*_ to a higher value. Nevertheless, given that the sensing pH range of an indicator usually spreads over p*K*_*app*_ ± 1, all described materials are applicable for physiological sensing applications, especially for epidermal monitoring. The skin surface pH normally ranges between 4.2 and 6.1 [17], whilst pH of sweat covers the range 5.2 to 6.2, and in some cases can increase up to 7.3 during exercise possibly due to migration of ammonia from plasma to sweat [48]. We have previously reported that the cotton materials based on the same indicator dye when tested against temperature changes in the range 20 °C to 40 °C showed negligible changes in their calibration plots [49].

Transparent cellulose films and paper materials have sufficient mechanical stability to be used in wearable application scenarios without additional modification. The response of both paper and cellulose films, was fully reversible with response times between 1 and 2 min measured in a simple cuvette test. Given their p*K*_app_ values, CF-1* and CP-1* show promise for colourimetric measurement of apparent skin surface pH. Additionally, the potential use of CF-1* in real skin tests was confirmed in artificial sweat solutions, Fig. S2, with the calibration plot covering the dynamic range of interest and which showed only a small shift in p*K*_app_ value from 6.2 to 6.3 compared to measurements in pH buffers. In contrast to self-standing materials, nanocellulose and microcellulose particles have to be dispersed in aqueous or polymer solution and deposited on a wearable substrate by an appropriate method, ideally compatible with mass production, i.e. inkjet printing, knife coating, screen-printing, etc. [50]. Since the colouration of nanocrystals was significantly less pronounced in comparison to functionalised microcellulose particles whose colouration and subsequent purification was also more facile**,** we selected MC − 1* particles for further characterisation and modification of textile materials.

### pH responsive polyester textile

Polyester textiles are commonly used in sports apparel and, unlike cotton fibres, these materials cannot be directly modified using simple vinylsulfonyl chemistry. For that reason an alternative method of textile functionalisation was performed: the prepared MC − 1* particles were embedded into binder monomers/polymers and coated onto polyester fabrics thus forming a pH-responsive smart textile. This fabrication procedure is completely compatible with high volume production techniques, such as screen printing, commonly used in the textile industry.

Initially, we evaluated binder monomer materials that are typically used by the textile industry for screen-printing coloured pigments onto textiles. The coloured microcellulose particles MC − 1* were mixed into the acryl-based binder monomer dispersion Printperfekt 226 EC. After applying the mixture onto a textile and heating to 130 °C for 20 min, a layer was obtained that showed an irreversible green colouration. We suppose that the acrylic acid formed during the curing process adjusts an acid pH in the coating which is not affected by different pH buffer solutions in contact with the textile. Similarly, coloured pH indicator particles cured into the polyurethane-based Printperfekt Base FF did not show any colour changes when changing the pH.

A more successful method was achieved by embedding MC − 1* into the medical-grade biocompatible hydrogel HydroMed™ D4 which was subsequently coated onto a polyester T-shirt. Hydrogel D4 was chosen as it is soluble in aqueous ethanol and can be dried at room temperature and because it is well-known to be highly ion-permeable [51]. However, the mechanical stability of D4 is inferior to the afore mentioned textile binder monomers, and after ten washings using a conventional alkaline washing agent at 40 °C, a 30% decrease in colouration of the smart textile was observed.

### Dynamic characterisation of MC − 1*/hydrogel@polyester

**Fig. 4:**
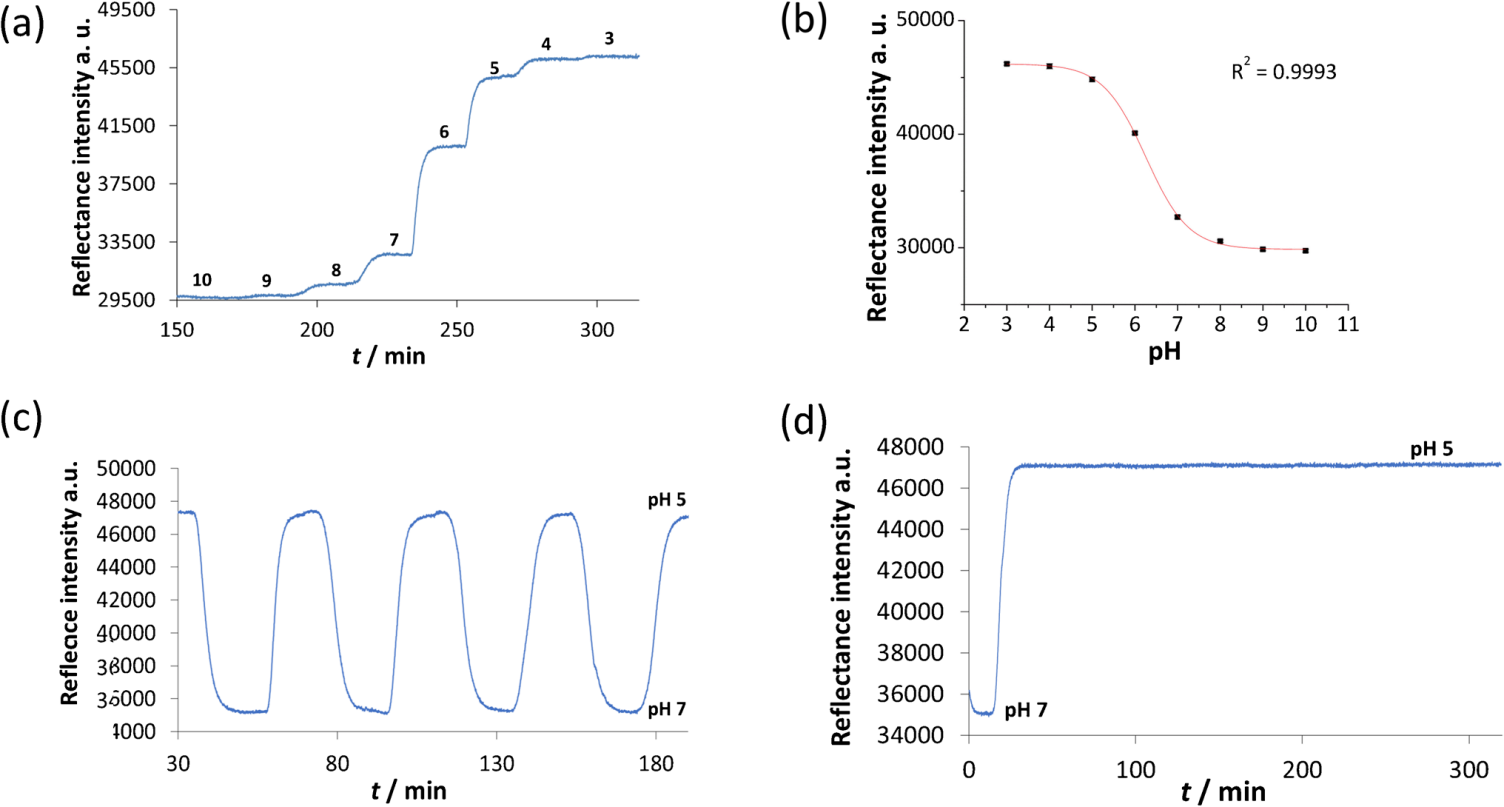
**a** Dynamic reflectance response of the MC − 1*/hydrogel@polyester to decreasing pH in a flow-through cell (pH 10 to 3). **b** pH response curve of the functionalised textile. **c** Reversibility between pH 5 and 7. **d** Reflectance of the textile during continuous 5-h exposure to a pH 5 buffer solution in a flow-through cell, after initial change from pH 7

The HydroMed™ D4-based sensor layers on polymer fabric were next evaluated in a flow-through cell to determine their dynamic sensing characteristics and evaluate the feasibility of integration into a wearable system. Since the textile is not transparent, a laboratory spectrophotometer with a reflectance probe was used for the characterisation. The reflectance response to pH values is shown in Fig. 4a. After a decrease in pH of 1 unit, the response stabilises after approximately 5 min on average (calculated as the time needed to reach 90% of the full step response).

A pH response curve was obtained by plotting the stabilised reflectance intensity in each buffer solution (*n* = 3) against pH of the buffer solution, Fig. 4b.

The expected sigmoidal relationship was obtained and a Boltzmann curve fitted to the experimental data with excellent correlation (*R*^2^ = 0.9993), thus yielding the following calibration function:

1 $$\mathrm{pH}=0.5007\left[\ln\left(\frac{<span class='convertEndash'>46188-29858</span>}{I-29858}-1\right)\right]+6.251$$

The relative standard deviation of the response was in all cases less than 0.5% (*n* = 3), which indicates good repeatability. The inflection point of the Boltzmann curve is the p*K*_app_ of the immobilised dye, and was found to be 6.25 — a good match with the p*K*_app_ values on cellulose film (6.1), Table 2. It has been documented elsewhere that immobilisation in polymer matrices can considerably affect the dissociation of halochromic dyes, and that the supporting fibrous material also plays a role in this process [22]. The obtained p*K*_app_ in pH buffers is 6.25, and in artificial sweat solutions is 6.0 (Fig. S2c), showing that immobilisation of** 1** in HydroMed™ D4 and transfer to a polyester shirt did not significantly affect the acidity constant and the pH range of the dye. Sweat pH varies with body location, but is generally slightly acidic to neutral, with average values ranging from 5.2 to 6.2 [12, 52]. These values fall well within the dynamic range of our sensor, which therefore makes it suitable for use in epidermal sweat pH sensing.

The textile was exposed to increasing (3–10), decreasing (10–3), and again increasing (3–10) pH (Fig. S3). No hysteresis in the calibration was noticed upon reversing the order of buffer solutions from acidic to basic (Fig. S4).

### Reversibility and stability

Wearable dermal sensors should ideally be fully reversible if the aim is to accurately follow natural epidermal pH fluctuations in real-time over extended periods. Therefore to evaluate the reversibility, polyester textile sensors were exposed to alternating flow of pH 5 and pH 7 buffered solutions, Fig. 4c. The sensor was found to be reversible with good repeatability: the relative standard deviation was less than 0.14% in both pH 5 and 7 (*n* = 4). The response time needed to stabilise after a step change of 2 pH units was found to be around 7 min on average for decreasing pH. A slightly slower response (8.5 min on average) was observed for pH increasing by 2 units. Changes in sweat pH in excess of 2 pH units are not expected during exercise [34, 53, 54] so these response times and working ranges are compatible with normal physiological sweat response. Operational stability of the sensor layers was evaluated next by exposing the films to a continuous flow of buffer over an extended time period, Fig. 4d. After initial stabilisation, the reflectance signal changed less than 0.16% after 280 min. (4 h 40 min.) exposure. This duration covers most exercise bouts (including marathons) and the good stability further demonstrates the potential of this type of sensor in sport physiology monitoring.

### Integration with wireless data acquisition system

**Fig. 5:**
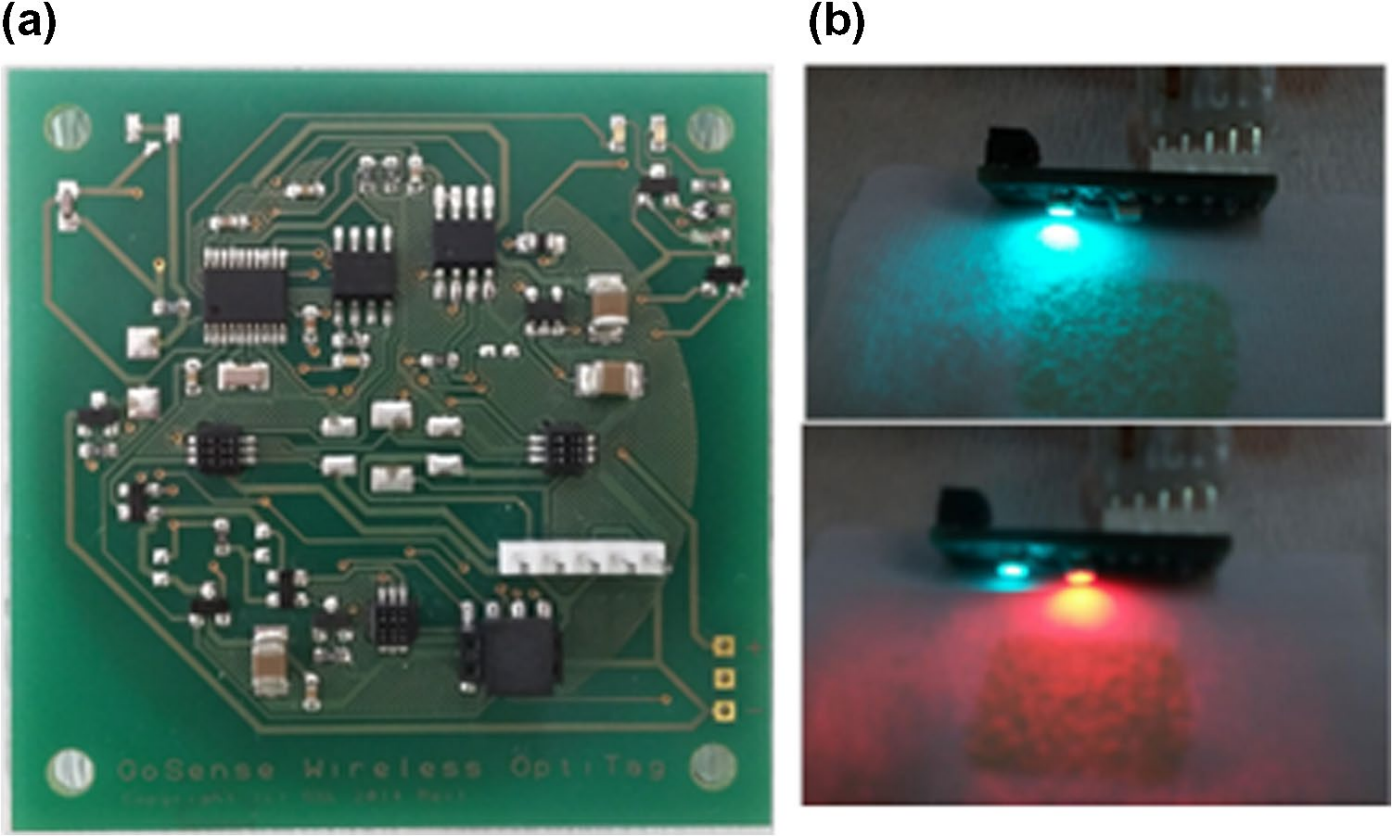
**a** Data logger electronics of the wearable sensor for operating the optoelectronic components, data acquisition and data transmission via NFC. **b** Cyan LED (*λ*_max_ = 502 nm), used as the primary light source for reflectance measurements from the textile, and a secondary red LED (*λ*_max_ = 634 nm) light source for referencing

After satisfactory results were obtained in the dynamic experiments, the textile was integrated with an ultra-low power data logger with miniature optical reflectance probe, to enable continuous monitoring of sweat pH. The data logger (6 × 6 × 1 cm) and probe (1 × 2 × 0.5 cm) are small and lightweight (17 g with coin cell), Fig. 5a. The data logger reads the halochromic textile with the reflectance probe. The probe consists of two optical components — an LED and a photodiode — organised in a planar configuration where a cyan LED with emission maximum at 502 nm is closely matched to the dye **1** absorption peak at 492 nm (Fig. 5b).

**Fig. 6:**
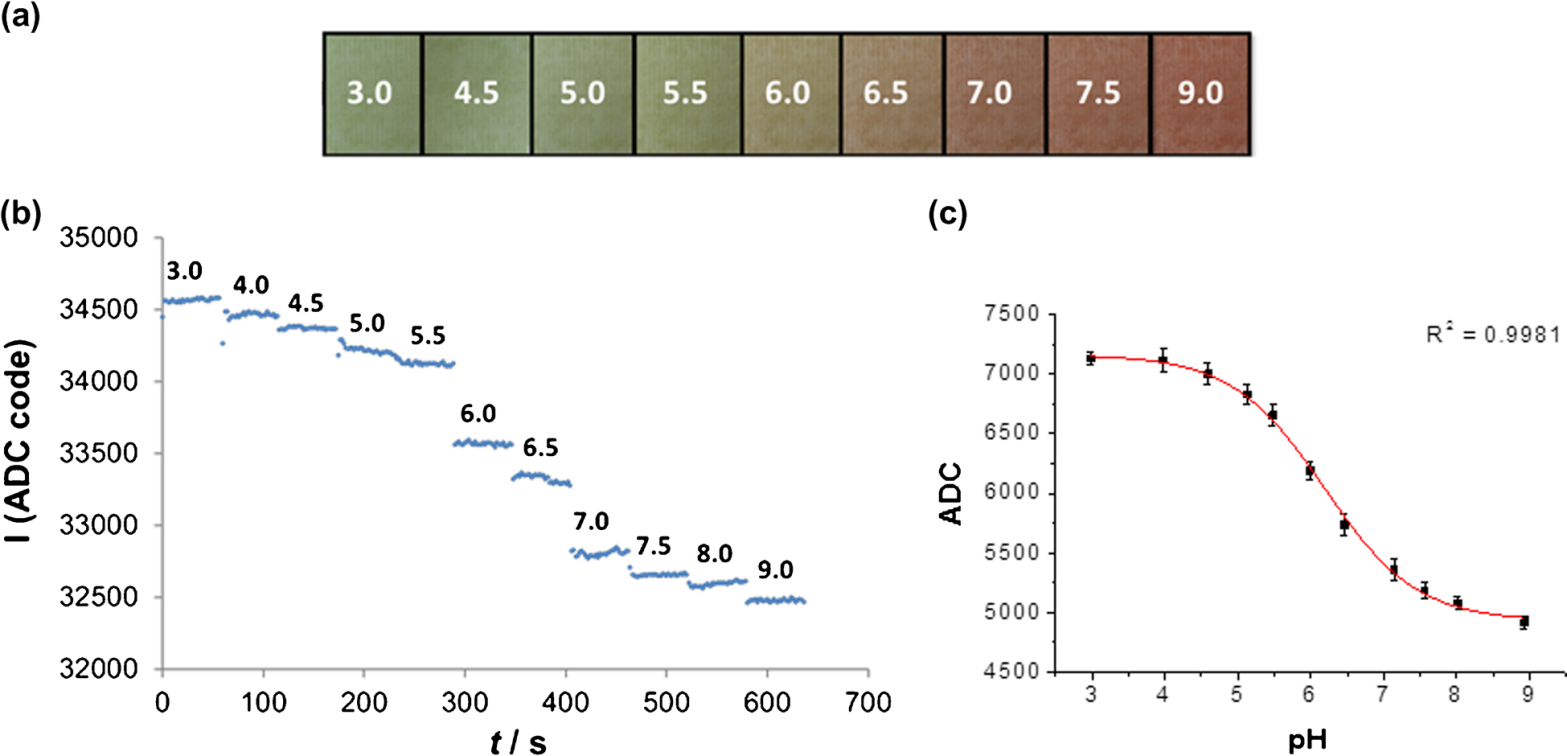
**a** Colour change of the MC − 1*/hydrogel@polyester sensor upon exposure to increasing pH of standard buffer solutions (pH 3 to 9). **b** Reflectance response recorded by the data logger and optical probe to the colour change. **c** Calibration curve after removal of ambient (background) light with fit to a Boltzmann function (red). Error bars represent one standard deviation (*n* = 4)

The photodiode detects reflected LED light from the halochromic textile. The greater the absorption by the textile at 502 nm, the less light of that wavelength comes back to the photodiode. Thus the photodiode response is inversely proportional to the pH (degree of deprotonation of **1**). The background light level is also recorded by the data logger and can be subtracted from the total reflectance signal to provide ambient light rejection and some motion artefact compensation. Reflectance data recorded by the logger is transferred via NFC to a smartphone using a custom made Android application. The total reflectance response to the pH-induced change in colour of the textile (Fig. 6a), expressed as arbitrary analogue-to-digital converter units (ADC), is shown in Fig. 6b.

The background light level was subtracted from the total response and a pH response curve was obtained by plotting the mean ADC value against the pH of buffer solutions. Four independent data sets (*n* = 4) were thus acquired, and the average values plotted against pH, Fig. 6c. As in the dynamic experiments, a Boltzmann curve was fit to the experimental data and the following calibration function was obtained (*R*^2^ = 0.9981):

2 $$\mathrm{pH}=0.6344\left[\ln\left(\frac{<span class='convertEndash'>7164-4929</span>}{\mathrm{ADC}-4929}-1\right)\right]+6.182$$

One standard deviation is shown with error bars in the figure (*n* = 4). *RSD* was in all cases better than 1.8%. The p*K*_app_ obtained as the inflection point of the fitted sigmoid function is 6.18. This is in good agreement with the p*K*_app_ = 6.25 obtained with the laboratory spectrometer, providing further evidence that the wearable system is suitable for vanguard epidermal pH monitoring. The pH range of the sensor is approximately from 4.5 to 7.5, with a quasi-linear response between pH 5 and 7.

### Analytical performance of the wearable system

**Fig. 7:**
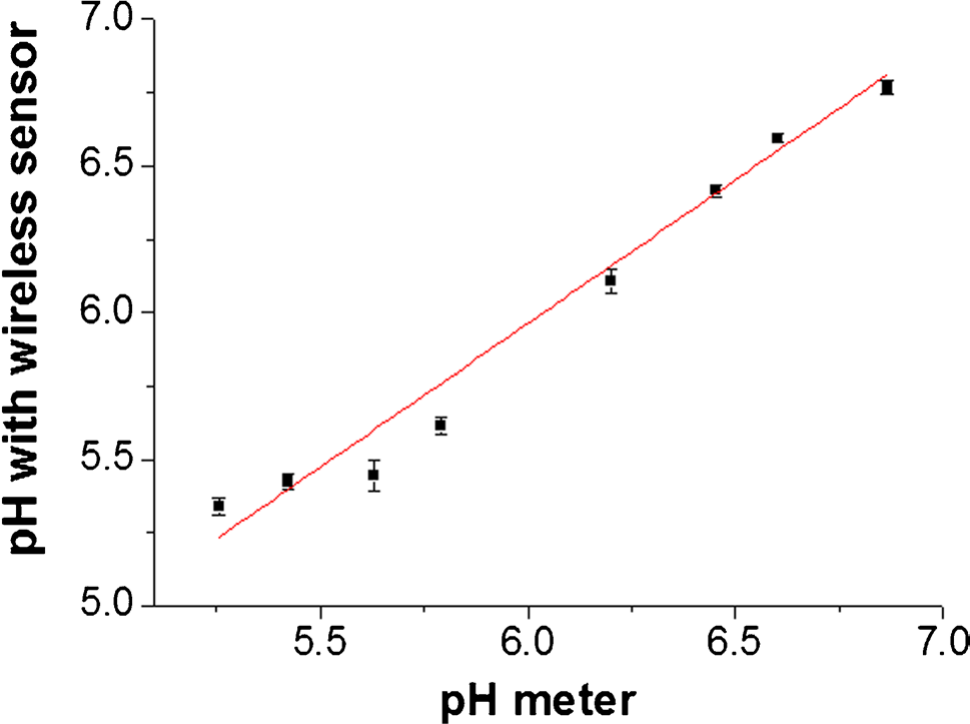
Correlation plot of pH determined with the wearable sensor against pH determined with the reference laboratory pH meter, a unity-slope line (y = x) is shown for reference, error bars represent one standard deviation around the mean (*n* = 6)

Finally, the analytical performance of the wearable system was evaluated by determining reliability parameters with eight unknown buffered samples. To simplify the calibration procedure, a three-point calibration function was constructed in the quasi-linear range (pH 5–7) which is the typical range for sweat. The pH was then calculated based on that calibration line: pH = 39,313 – ADC/892.8 (*R*^2^ = 0.9996). The pH values obtained with the wearable system using this calibration function were correlated against a laboratory pH meter, Fig. 7.

Each point in the correlation plot represents the average of six optical measurements, with the optoelectronic probe moved away from the textile between each measurement. The error bars represent one standard deviation. A unity slope line is shown on the plot for visualisation. The actual linear fit takes the following form: pH_wearable_ = 0.978 * pH_meter_ + 0.092, *R*^2^ = 0.9809, which indicates good agreement between measurements with the wearable system and the pH meter.

The measurement accuracy can be expressed as the difference between pH determined with the wearable system and reference pH meter, whilst precision is expressed as standard deviation around the mean, Table S1. The pH value can be determined with a precision better than 0.05 pH units and accuracy of 0.18 pH units in the quasi-linear range tested. For measurement outside the typical range of pH 5–7, the full Boltzmann curve should be taken for calibration.

This study demonstrates novel biocompatible, non-toxic and fully reversible pH sensitive materials and their performance in vitro with pH buffers and artificial sweat solutions, in combination with a miniature wireless data acquisition system. Unlike similar immobilisation chemistries demonstrated on cotton [28, 29], our approach is applicable on polyester materials and has proven non-toxicity, albeit with reduced durability and increased response times (7 min on average for step change of 2 pH units). A cotton-based, fully integrated wearable system for sweat pH monitoring — the microfluidic cloth analytical device (μCAD) [37] — demonstrated applicability in pH range 6 to 8, with a precision at different pH values of 3.6 to 6.0%. The practical challenge of *on-body* sweat sampling has been addressed with microfluidic sweat-wicking wearable patches [30], chrono-sampling [31] and screen printed biocompatible microfluidic systems [32]. However, irreversible chemistry and discrete-time signal analysis (digital colourimetric analysis with smartphone/flatbed scanner images) make these systems less applicable for continuous monitoring.

Here, the reversibility and stability of our new materials and their halochromic response when tested in a laboratory setting, at high fluid flow-rates, demonstrates satisfactory robustness for future use in real sweat monitoring. Following the recommendations and guidelines of the ongoing IUPAC project [55], the next challenging step in development of fully wearable monitoring devices is the integration of continuous microfluidic sampling and tests with real sweat.

## Conclusions

We have produced a diverse set of halochromic and biocompatible materials that exhibit stable and reversible response in the physiological range, making them ideally suited for real-time epidermal pH monitoring. The simple yet robust and scalable covalent immobilisation by vinylsulfonyl conjugation of an azo pH indicator dye has been employed to functionalise different commercially available cellulosic materials (nanocrystals, microcellulose particles, transparent cellulose films and cellulose paper). The subsequent UV–Vis spectroscopic characterisation of the modified materials revealed a highly efficient dyeing procedure in all material types, with the exception of nanocrystalline cellulose whose colouration intensity was found to be lower in comparison with functionalised microcellulose particles. The apparent p*K*_app_ of the immobilised azo indicator dye was found to depend upon the type of pristine cellulosic material, and ranged from 5.5 for cellulose paper to 6.9 for nanocellulose particles. The dynamic measurement range of the sensing materials exceeds p*K*_*app*_ ± 1 pH unit making them compatible with the physiological epidermal pH range. Moreover, the transparent cellulose films and opaque paper materials have sufficient mechanical stability for use directly on skin as wearable patches without additional modification, albeit missing the flexibility of textile materials. The response of both paper and cellulose films was fully reversible with response times of between 1 to 2 min. Their respective p*K*_app_ make them suitable for the colourimetric determination of *apparent skin surface pH*. If applied directly on skin, halochromic transparent cellulose films will also allow exchange of optical signals through the skin. This property make them promising candidates in the development of multimodal optical wearable sensors that combine monitoring of physical parameters, like heart rate, together with chemical biomarkers on a single wearable optoelectronic platform.

A polyester sports textile was functionalised with the biocompatible hydrogel containing halochromic cellulose microparticles. This is a scalable fabrication process compatible with screen printing for high volume production. The pH response of the smart textile was found to be fully reversible in the range 5–7 pH units (*RSD* = 0.14%), and highly stable over a period of more than 4 h exposure to continuous flow of buffer (signal change of 0.16%). We finally demonstrated the potential of this smart textile for effective real-world performance by combination with a low-power wearable data logger and reflectometer probe – achieving real-time pH tracking over a wide dynamic pH range (3–9 pH units) and with a greatly simplified linear calibration for the physiologically relevant epidermal pH range (5–7 pH units) with an accuracy of 0.18 pH and precision of 0.05 pH units.

## Supplementary Information

The online version contains supplementary material available at XXXX.

Below is the link to the electronic supplementary material.

ESM 1 (PDF 542 KB)

## Data Availability

No datasets were generated or analysed during the current study.
