# Supplementary material for: Design of halochromic cellulosic materials and smart textiles for continuous wearable optical monitoring of epidermal pH
Source: Mikrochim Acta. 2025 Jun 4;192(7):405. doi: 10.1007/s00604-025-07259-x (PMC12137401; doi:10.1007/s00604-025-07259-x)
Supplement: Supplementary file 1 — (PDF 542 KB) [file 604_2025_7259_MOESM1_ESM.pdf]

Supplementary Material

Design of halochromic cellulosic materials and smart textiles for continuous wearable optical monitoring of epidermal pH

Gerhard J. Mohr[1*], Petar Kassal[2], Iva Žuvić[2], Krzysztof K. Krawczyk[1], Matthew D. Steinberg[3], Ivana Murković Steinberg[2*]

[1] Joanneum Research Forschungsgesellschaft mbH – Materials, Franz-Pichler-Straße 30,Weiz A-8160, Austria

[2]University of Zagreb Faculty of Chemical Engineering and Technology, Marulićev trg 19, HR-10000 Zagreb, Croatia

[3]GoSense Wireless Ltd., Cambridge, CB23 6FN, UK

*corresponding authors

Contents

Fig. S1 Visual (a), and UV-Vis absorbance spectral changes (b), of functionalised nanocellulose NC−**1** dispersed in buffer solutions of different pH values

Fig. S2 (a) Spectroscopic pH titration - absorption spectra of transparent cellulose film CF−**1*** in artificial sweat solution and respective pH titration plots and calculated pK_{app} values for **(b)** CF−**1*** and **(c)** MC−**1***/hydrogel@polyester

Fig. S3 Reflectance intensity at 525 nm of the smart textile exposed to buffer solutions with increasing pH (3 – 10), decreasing pH (10 – 3), and again increasing pH (10 – 3)

Fig.S4 Three calibration curves with Boltzmann fit, constructed from reflectance data in Figure S3

Table S1. Precision (standard deviation) and accuracy (error) of the wearable sensor in pH units, *n* = 6

(a)

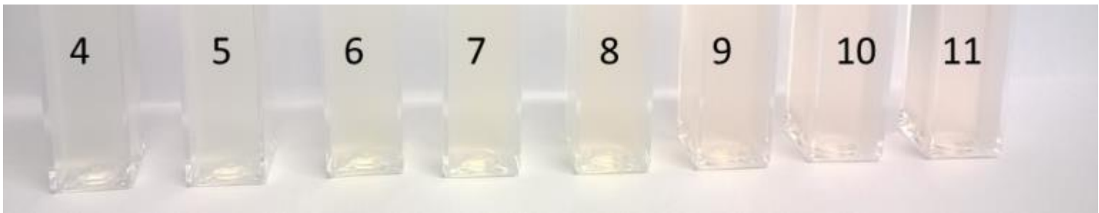

(b)

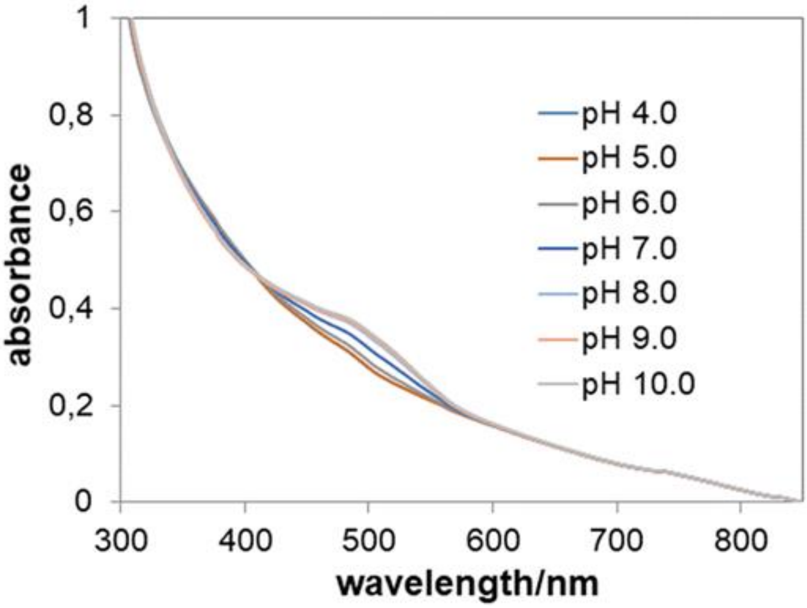

Fig. S1 Visual (a), and UV-Vis absorbance spectral changes (b), of functionalised nanocellulose NC−**1** dispersed in buffer solutions of different pH values

(a)

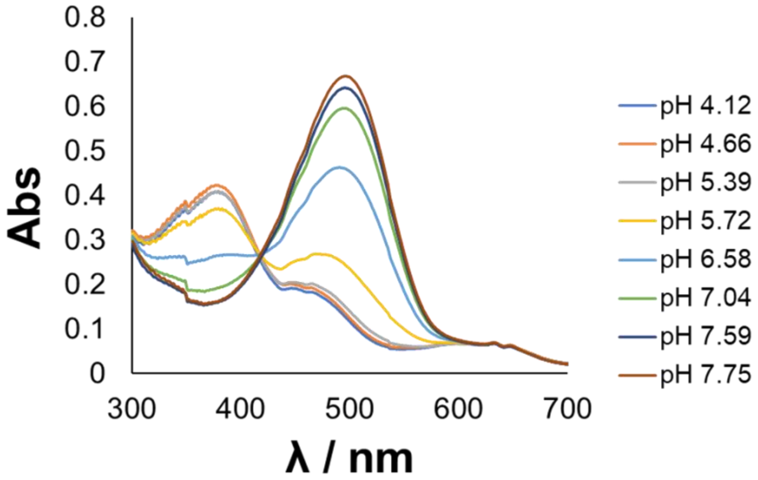

(b)

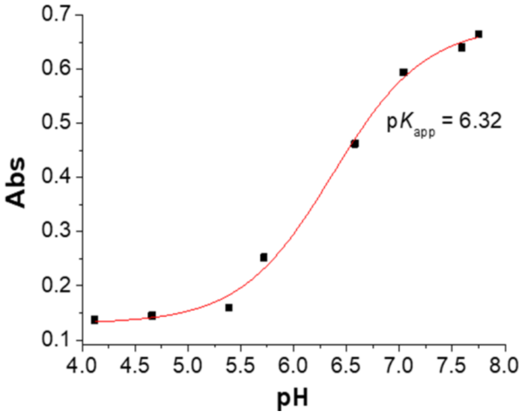

(c)

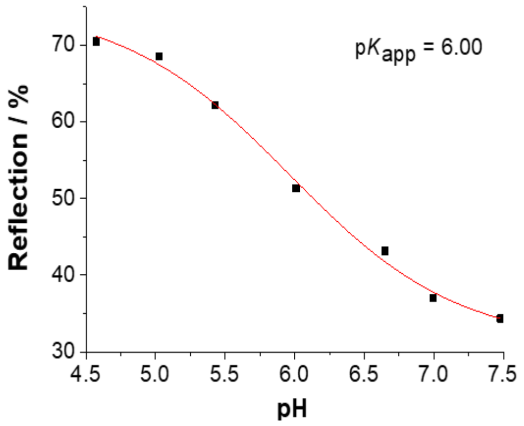

Fig. **S2** **(a)** Spectroscopic pH titration - absorption spectra of transparent cellulose film CF–**1*** in artificial sweat solution and respective pH titration plots and calculated pK_{app} values for **(b)** CF–**1*** and **(c)** MC–**1***/hydrogel@polyester

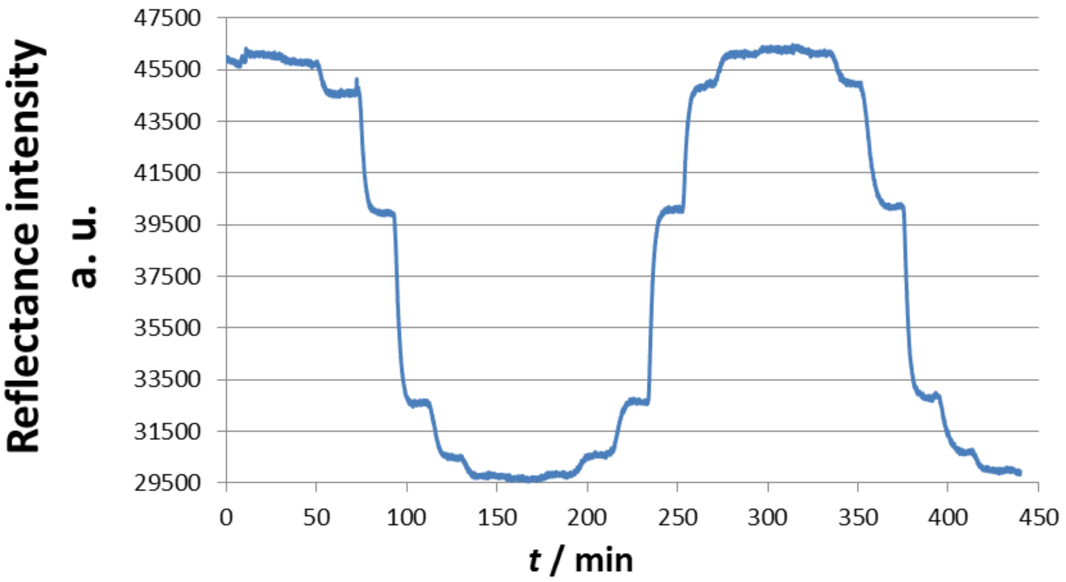

Fig. S3 Reflectance intensity at 525 nm of the smart textile exposed to buffer solutions with increasing pH (3 – 10), decreasing pH (10 – 3), and again increasing pH (10 – 3).

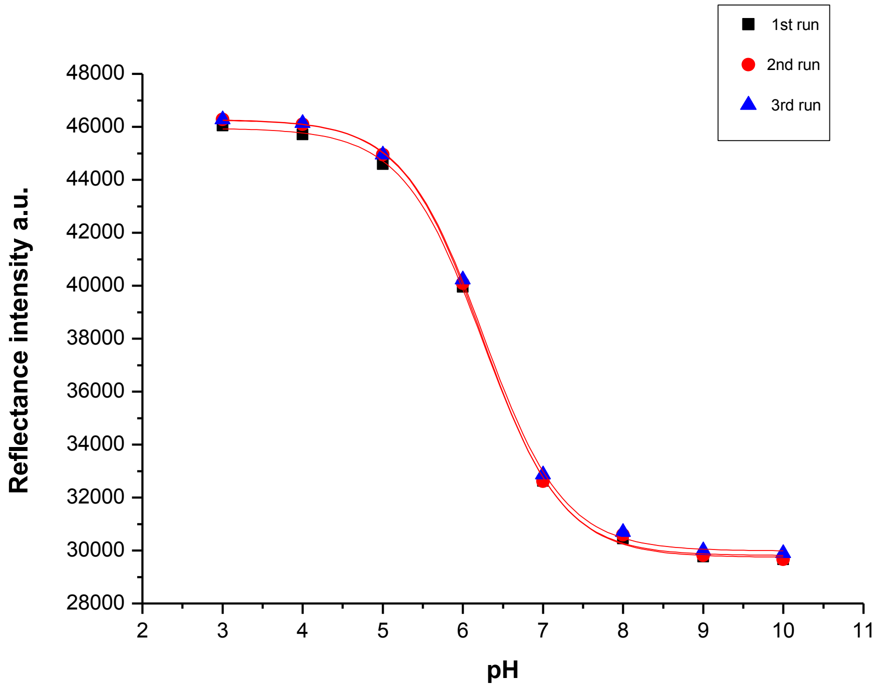

Fig.S4 Three calibration curves with Boltzmann fit, constructed from reflectance data in Figure S3.

Table S1. Precision (standard deviation) and accuracy (error) of the wearable sensor in pH units, n = 6

pH meter	pH with wearable sensor	precision	accuracy
5.26	5.34	0.03	0.08
5.43	5.43	0.03	0
5.63	5.45	0.05	-0.18
5.79	5.61	0.03	-0.18
6.20	6.11	0.04	-0.09
6.45	6.41	0.02	-0.04
6.60	6.59	0.02	-0.01
6.87	6.77	0.02	-0.10
